# Early Detection for Better Patient Outcome: A Case Report on Two Patients Presenting With Fibrodysplasia Ossificans Progressiva at Tikur Anbessa Specialized Hospital, Ethiopia

**DOI:** 10.1155/cro/2161762

**Published:** 2025-01-29

**Authors:** Alazar M. Haile, Abrham W. Azale, Birhanu Ayana

**Affiliations:** Tikur Anbessa Specialized Hospital, Department of Orthopaedics, College of Health Sciences, Addis Ababa University, Addis Ababa, Ethiopia

## Abstract

Fibrodysplasia ossificans progressiva is an ultrarare disorder of endochondral ossification. It is unfamiliar to most care providers in low-income countries such as Ethiopia. Even though the clinical presentation is typical, most cases remain misdiagnosed in our region. Moreover, we hypothesize that many such cases undergo unnecessary or harmful interventions for a painless lump. In Ethiopia, with a population of approximately 120 million, only one case has been reported in the literature so far. We present two cases that were referred to our institution for a biopsy of a mass. This report is aimed at summarizing the typical presentation of the disease and at highlighting the harmful interventions one should avoid in such patients. We also hope that this report serves as an entry point to try to find more patients with this similar condition early in their clinical course. Furthermore, we believe that in order to lessen the overall impact of the illness, it is crucial to provide caregivers with health education about the causes of disease flare-ups.

## 1. Introduction

Fibrodysplasia ossificans progressiva (FOP), an ultrarare disorder, is a severe form of heterotopic ossification (HO) which leads to progressive bone formation in extraskeletal tissue impairing mobility and affecting the patient's quality of life [1]. It is also described as myositis ossificans progressiva, Munchmeyer disease, or stone man syndrome [2]. It usually occurs due to a mutation in the Activin A receptor Type 1 (ACVR1) leading to dysregulated bone morphogenetic protein (BMP) signaling [3]. The dysregulated BMP signaling promotes endochondral bone formation in muscles, tendons, and ligaments [1]. The global prevalence has been estimated between 1/1,200,000 and 1/2,000,000 [4]. So far, only 37 cases have been reported in Africa [5]. In the published records, only one case was reported in Ethiopia by Solomon et al. back in 2018 [6]. Diagnosis is mostly clinical based on the constellation of symptoms of bilateral great toe malformation, progressive HO in characteristic anatomic pattern, and episodic flareups [7]. Locally, most health professionals are unaware of the condition, predisposing these at-risk groups to potential harm from “innocuous” procedures like biopsy. Treatment is generally symptomatic with a focus on avoidance of factors exacerbating flare-ups [8]. We present two cases, a 7-year-old boy and a 11-year-old girl, who were referred to our unit at Tikur Anbessa Hospital as lumps for possible histopathologic diagnosis. This case report has been reported in line with the Surgical CAse REport (SCARE) criteria of 2020 [9].

## 2. Case Presentation

### 2.1. Case 1

A 7-year-old boy from the central part of Ethiopia, with unremarkable prenatal history and normal developmental milestones, presented with difficulty to sit due to stiffness. Associated with this, he has multiple soft tissue “lumps” and bilateral great toe deformity. The toe deformities were first noticed at birth by the parents. It was asymptomatic, and no measures were taken at the time. The patient was relatively well until 2 years back when he began to experience a soft tissue “lump” that first appeared over the scalp. Subsequently, multiple other “lumps” appeared on the neck, subsequently on the back, and now involve the lower extremities as well. Some of these “lumps” regress on their own, but most harden with time. Currently, he has difficulty in squatting affecting his ability to use the toilet. He was seen at multiple health facilities and was subsequently referred to our care for biopsy and histopathologic diagnosis. The parents did not recall a history of trauma preceding his symptomatology.

**Figure 1:**
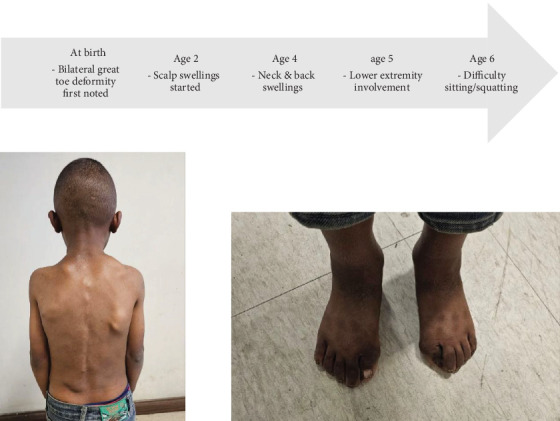
Multiple swellings over the dorsum of the trunk and bilateral hallux valgus.

**Figure 2:**
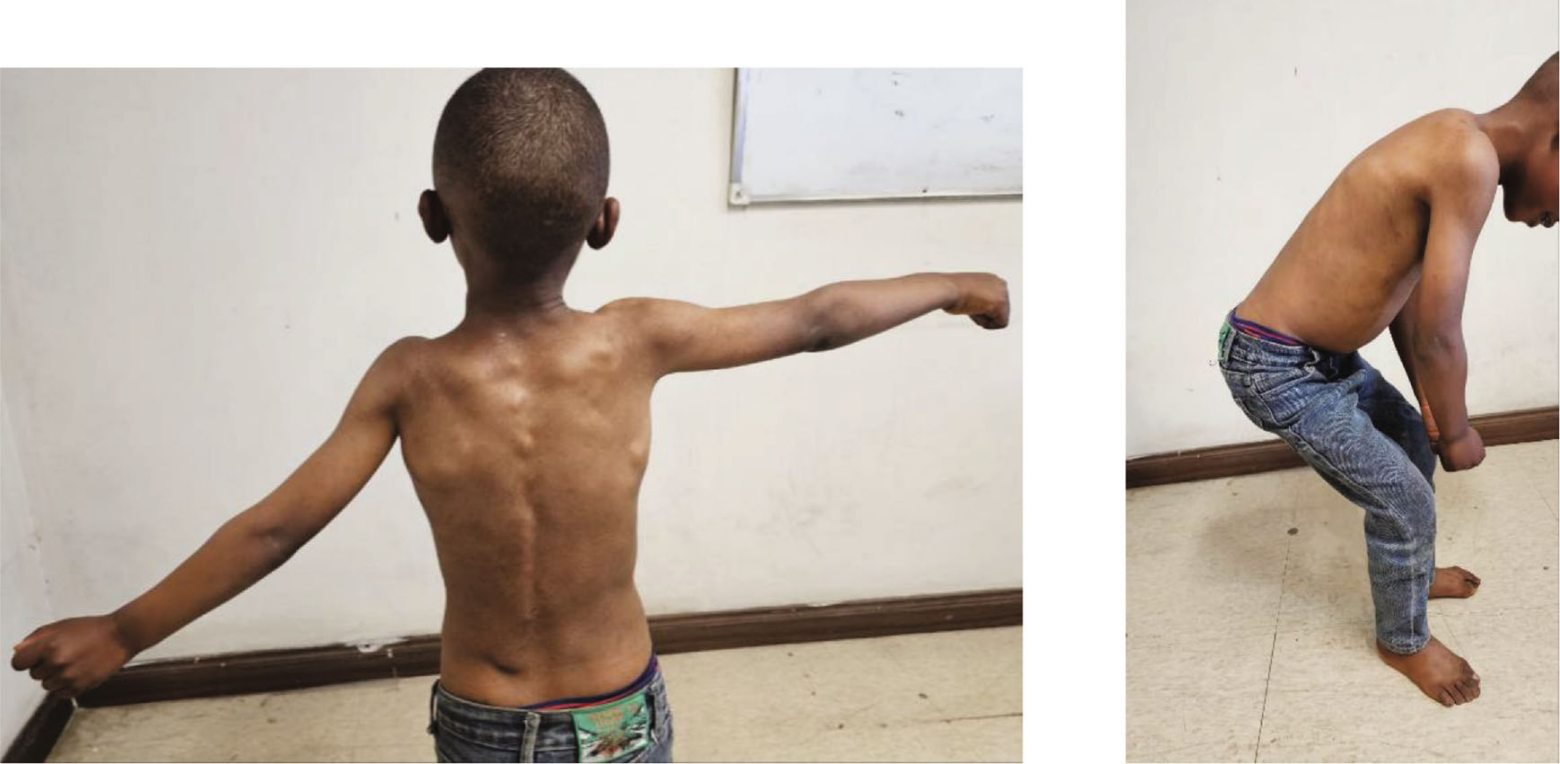
Inability to elevate the left arm above his shoulders. The picture also shows the patient having difficulty with squatting.

On exam, the patient has multiple fixed, nontender, and bony hard masses of variable sizes all over the body. There is also bilateral valgus deviation of the great toes, as illustrated in Figure 1. Neck and hip range of motion as well as overhead arm elevation is restricted, as shown in Figure 2. He has a cumulative analogue joint involvement scale (CAJIS) score of 8.

Routine laboratory tests such as complete blood count, organ function tests, and serum electrolyte were within the normal limits.

**Figure 3:**
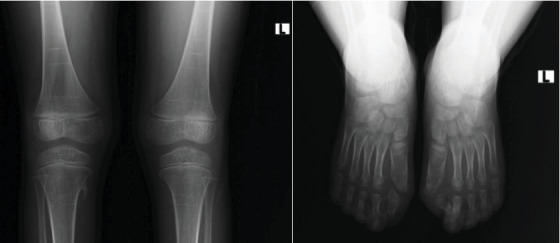
Knee x-ray shows a pedunculated bony outgrowth from the medial side of the right proximal tibia. Foot AP x-ray shows bilateral hallux valgus with macrodactyly.

The knee radiograph shows right side bony outgrowth from the medial aspect of the proximal tibia. The lesion has medullary and cortex continuity with the tibia (refer to Figure 3). Foot radiographs show bilateral hallux valgus with a hallux valgus angle of 50° and a first intermetatarsal angle of 12° on both feet (see Figure 3). Additionally, the head of the first metatarsal is wedge shaped bilaterally with the right side having a small proximal phalanx.

**Figure 4:**
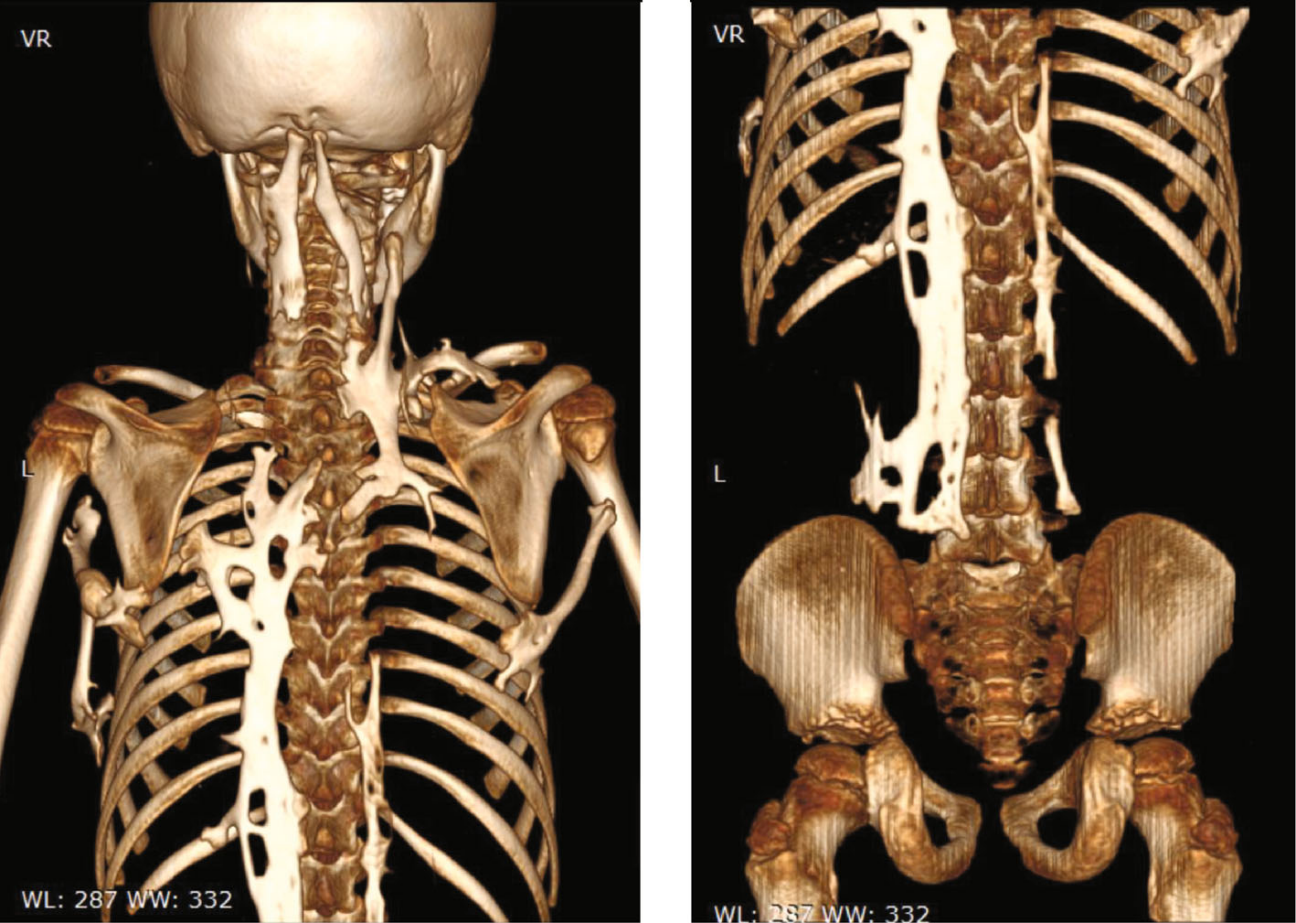
Whole-body CT shows sheets of extraskeletal ossification along the prevertebral soft tissue from the occiput to the sacrum. There are also bony bridges from the ribs to the respective humeri.

Whole-body CT (see Figure 4) shows midline long-segment extensive ossification of the posterior paravertebral soft tissue with vertical bony bridges spanning from the occipital protuberance to L4 vertebrae. There is also a bony bridge extending from the right ninth and left eighth rib to the respective humeri. Additionally, there is also a superior bony projection in the soft tissue extending from the superior aspect of the right scapula to the right lateral neck region. Bilateral femoral necks are also short and broad.

After a multidisciplinary discussion between the orthopedic oncology and radiology unit, it was decided to follow the patient conservatively and avoid potential triggers including biopsy. The family was advised on the disease course and preventative measures to limit further flare-ups. Supportive genetic test was not done due to lack of availability.

### 2.2. Case 2

An 11-year-old girl from Eastern Ethiopia presented with neck and elbow stiffness of 2-year duration. Associated with this, she has multiple soft tissue swellings over her trunk and bilateral great toe deformities. Toe deformities were initially noticed at birth and further medical help was not sought. Multiple soft tissue lesions initially appeared over the trunk and neck by the age of 6 months which subsequently “dried up” as explained by her parents. They also began to notice restriction of her neck movement by the age of 2. She has visited nearby health centers and has received multiple injections for pain relief with each bout. She was later referred to the orthopedic oncology unit at Tikur Anbessa Hospital for biopsy and further management. Otherwise, she has no family history of similar complaints. She had an uneventful prenatal history. Age-appropriate developmental milestones were achieved during infancy and early childhood. She is fully vaccinated per our local vaccine protocols.

**Figure 5:**
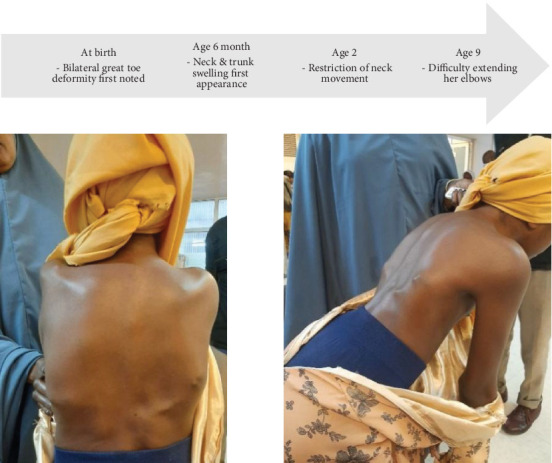
Multiple lumps visible over the back. Restriction of forward flexion of the trunk also noted.

**Figure 6:**
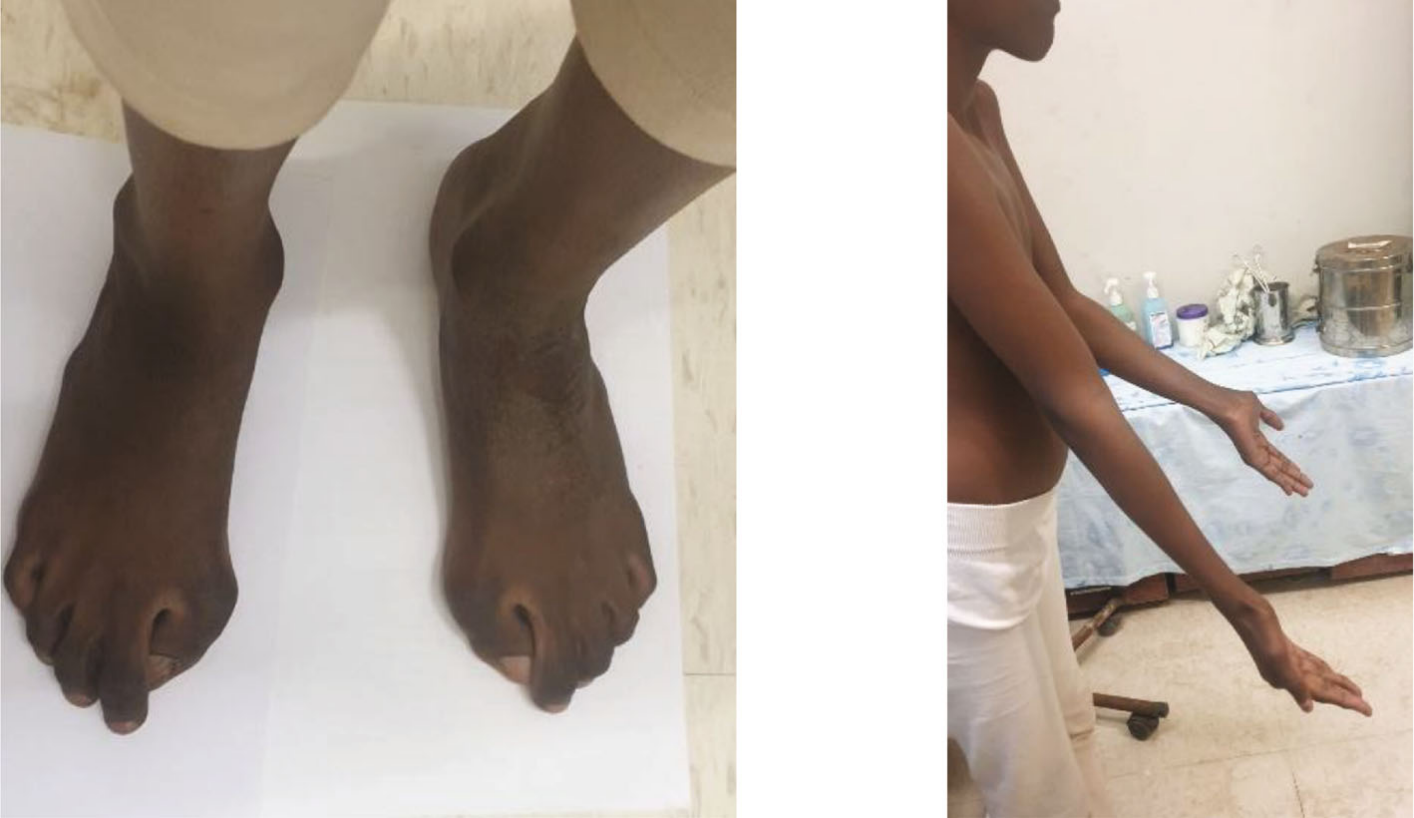
Bilateral hallux valgus and fixed flexion deformity of the left elbow.

On exam, she has multiple firm, nontender swellings all over the body, as depicted in Figure 5. She also has bilateral hallux valgus, lateral deviation of the trunk, restricted neck range of motion, and fixed flexion deformity of the left elbow, as illustrated in Figure 6.

The complete blood count and basic metabolic profiles (organ function tests and serum electrolytes) were within normal ranges.

**Figure 7:**
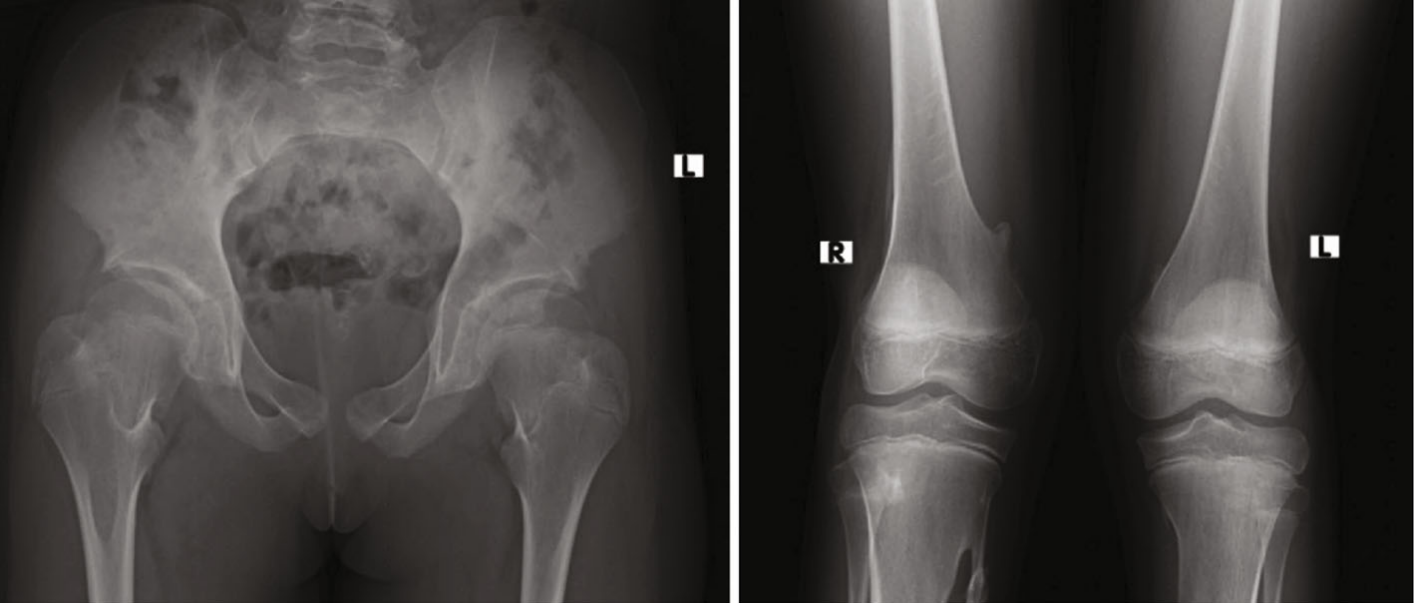
Pelvis AP x-ray shows bilateral short broad femoral necks. Knee x-ray shows bony outgrowth from the right distal femur and proximal tibia.

**Figure 8:**
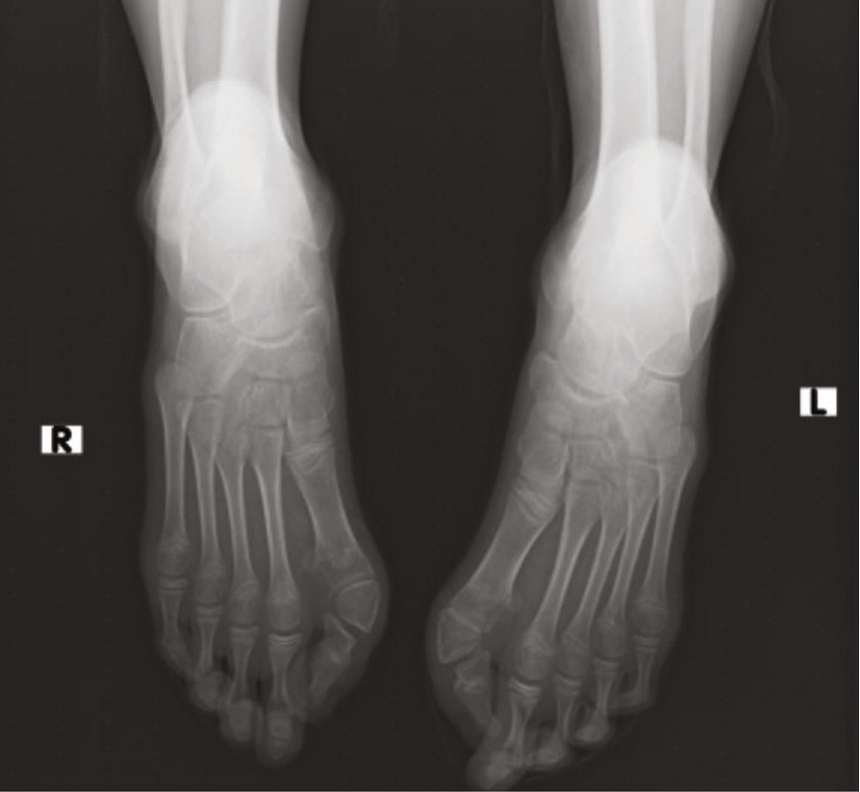
Foot AP x-ray shows bilateral hallux valgus with wedging of the first proximal phalanges.

**Figure 9:**
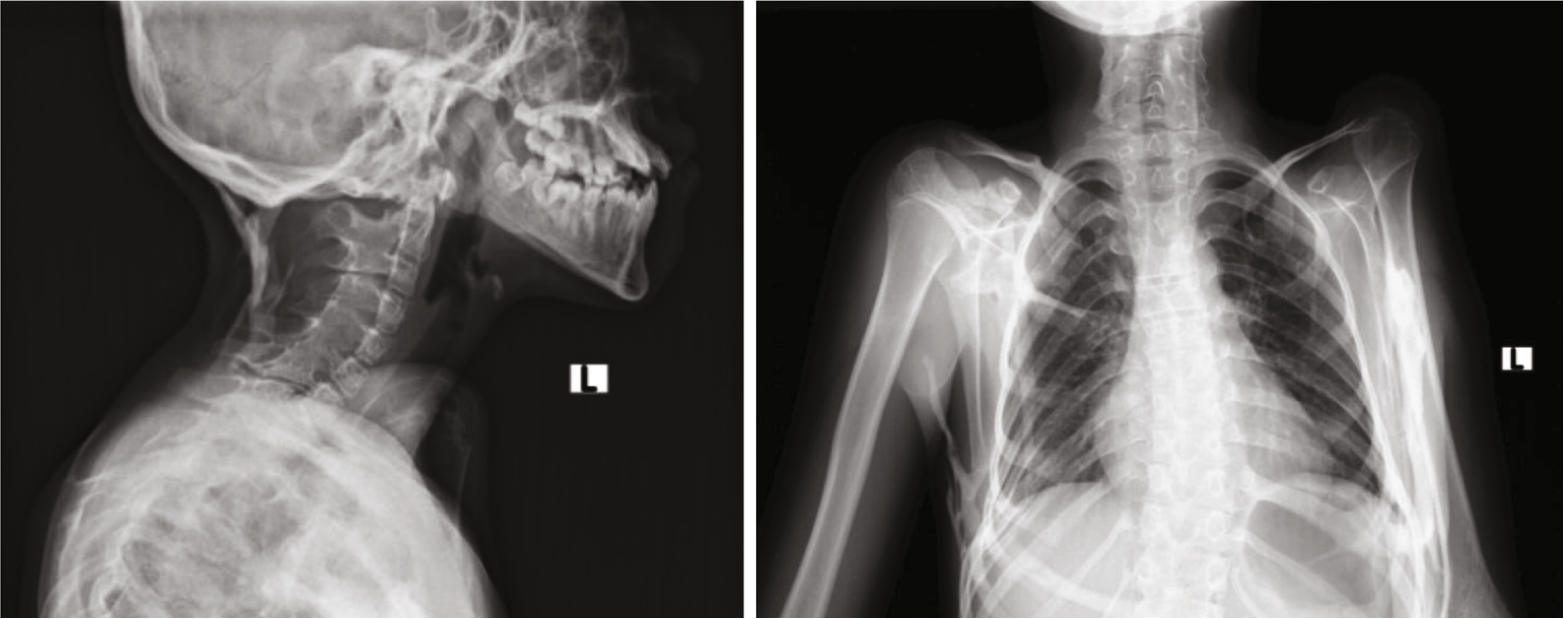
Lateral cervical x-ray shows band-like ossification over the posterior paracervical soft tissue. Chest x-ray shows bony bridges connecting the right and left humeri with the axial skeleton.

The hip radiograph shows bilateral short and broad femoral necks (refer to Figure 7). The knee radiograph shows pedunculated bony outgrowth from the right medial distal femur and proximal tibia which has medullary continuity (see Figure 7). Foot radiographs show bilateral hallux valgus with wedge-shaped proximal phalanges (see Figure 8).  Figure 9 illustrates the ossification of the posterior paracervical soft tissue along with bridging ossification that connects the upper extremities to the axial spine.

**Figure 10:**
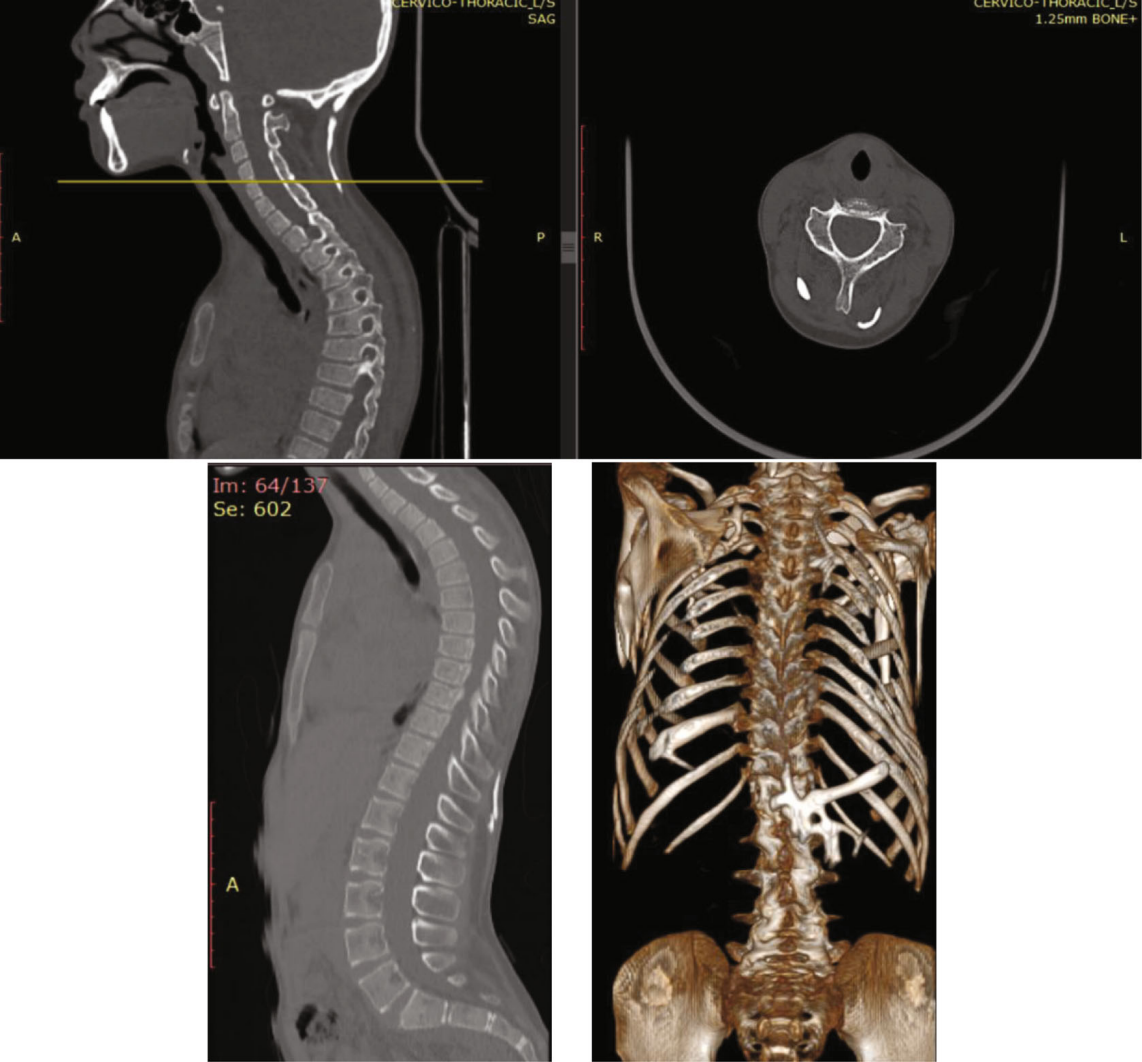
Whole-body CT shows ossification of the posterior paravertebral soft tissue, and thoracic kyphosis is depicted.

Whole-body CT shows extensive long band-like intramuscular ossifications that attach to the occiput and extend down to the lumbar area. Extensive ossifications are also present around the shoulder and arm bilaterally (see Figure 10). Sagittal cuts show thoracic kyphosis.

A genetic test was not done due to lack of availability. Her parents were educated on the disease process, its course, and ways of preventing further flare-ups.

## 3. Discussion

FOP is amongst the rarest disorders of primates described as early as 1692 by Patin [10]. It presents at birth with bilateral malformation of the big toe usually a hallux valgus [11]. On average, by the age of five, episodic painful flare-ups occur either induced or spontaneously which subsequently mature into extraosseous bone formation in a distinct anatomic pattern progressing from dorsal, axial, and proximal locations to ventral, appendicular, and distal locations [12, 13]. Both of our patients presented with the typical clinical features which was also supported by radiographic studies as outlined in the case descriptions above. In the later stages of the disease, it inevitably involves major joints of the axial and appendicular skeleton leading to bony ankylosis [14]. Most patients became wheelchair-bound by the age of 30 [14]. And mortality is usually due to thoracic insufficiency syndrome in the later stage of the disease [15]. The diagnosis is clinical; however, genetic confirmation can be carried out whenever available [7]. The differential diagnosis includes progressive osseous heteroplasia and Albright hereditary osteodystrophy [16]. But these can be ruled out by the location of the ossification and lack of predictable pattern of progression.

Although trauma is usually the most common inciting factor of the flare-ups, they can follow a multitude of iatrogenic interventions [3, 17]. Nearly 90% of FOP patients worldwide are initially misdiagnosed [17]. The soft tissue ossifications can be confused with a malignancy to the uninitiated eye. Approximately, 67% of them undergo unnecessary diagnostic procedures that leads to permanent loss of mobility and acceleration of the disease process [17]. Time to accurate diagnosis is also delayed in the majority leading to a delay to appropriate care [7]. Both of our patients presented late with a mean delay in diagnosis of 9 years, and the reason for referral in both cases was for biopsy. This shows a gap in the awareness and identification of the disease process calling for a more proactive measure to educate physicians who may be involved in the care of these patients. Even though the disease entity is somewhat rare, clinical diagnosis is straightforward if one knows what to look for. For example, they have started a newborn screening program to identify these cases early by looking at the great toes in Brazil [18]. In our setup, at the very least, we urge that all care providers should be aware of this condition and identify it whenever it occurs based on its typical phenotypic presentation. We also urge to streamline these patients, if possible, to an appropriate focal person for individualized care.

The medical treatment of FOP generally involves managing acute flare-ups and slowing down the disease progression [19]. During acute flare-ups, the goal is to limit inflammation, which is the main driving factor. These flare-ups are treated with a short course of NSAIDs or high-dose corticosteroids [20]. Corticosteroids are most effective when administered early and should generally be avoided for flare-ups involving the neck or trunk [20]. They can also be used prophylactically following a significant soft tissue trauma or perioperatively [20]. However, these agents are not suitable for long-term use as their toxicities increase over time. In cases where corticosteroids are ineffective, bisphosphonates have also been tried with some success, although they have not been shown to prevent future flare-ups [20].

Significant strides have been made in the understanding of FOP at a molecular level, laying the foundation for potential medications to slow down the disease progress. These drugs, currently undergoing clinical trials, target various checkpoints on the Activin A–induced BMP signaling pathway. These checkpoints include the Activin A substrate, the receptor ALK2/ACVR1, and downstream transcriptional effectors [21]. Garetosmab is a human anti-Activin A–neutralizing antibody that acts as a decoy receptor by binding to the substrate [21]. Saracatinib and zilurgisertib are selective ALK2 inhibitors that block the receptor [21]. Rapamycin, a mammalian target of rapamycin (mTOR) protein kinase inhibitor, and palovarotene, a nuclear retinoic acid receptor-ɣ agonist, inhibit chondrogenesis downstream [21]. Notably, palovarotene has been found to reduce the percentage of FOP patients that developed new HO as well as lower the volume of new HO at flare-up regions [22]. It is currently the only drug to have completed a Phase 3 clinical trial (MOVE trial, NCT03312634) [23]. Inflammation has also been explored as a therapeutic target. Inhibitors of the JAK/STAT signaling pathway, such as tofacitinib, have been shown to reduce the frequency of flare-ups and enable steroid-free remission [19]. In Africa, there are currently two ongoing clinical trials. The PROGRESS trial (NCT05090891) is a Phase 2 clinical trial investigating zilurgisertib and is actively enrolling patients (NCT05090891) [24]. The OPTIMA trial (NCT05394116) is another Phase 2 clinical trial investigating garetosmab (NCT05394116) [25]. Unfortunately, the expenses associated with travel and accommodation have posed a challenge, discouraging our patients from participating in these trials.

### 3.1. Limitations

Genetic confirmation was not done in our cases due to lack of availability.
